# Exploring Cognitive Changes in High-Risk Cardiac Patients Receiving Dexmedetomidine and Evaluating the Correlation between Different Cognitive Tools: A Cohort Study

**DOI:** 10.31083/j.rcm2508273

**Published:** 2024-08-01

**Authors:** Noor Anisah Abu Yazit, Norsham Juliana, Kamilah Muhammad Hafidz, Nur Adilah Shuhada Abd Aziz, Sofwatul Mokhtarah Maluin, Sahar Azmani, Nur Islami Mohd Fahmi Teng, Srijit Das, Suhaini Kadiman

**Affiliations:** ^1^Faculty Medicine and Health Science, Universiti Sains Islam Malaysia, 71800 Nilai, Negeri Sembilan, Malaysia; ^2^Department of Anaesthesia and Intensive Care Unit, Institut Jantung Negara, 50400 Kuala Lumpur, Malaysia; ^3^Research Management Centre, KPJ Healthcare University, 71800 Nilai, Negeri Sembilan, Malaysia; ^4^Faculty of Health Science, Universiti Teknologi MARA, 42300 Puncak Alam, Selangor, Malaysia; ^5^Department of Human & Clinical Anatomy, College of Medicine & Health Sciences, Sultan Qaboos University, 123 Muscat, Sultanate of Oman

**Keywords:** cognitive dysfunction, postoperative cognitive dysfunction, MMSE, high-risk patients, neuropsychological tests, cardiac surgery

## Abstract

**Background:**

Mini-mental State Examination (MMSE) is widely accepted clinically for postoperative cognitive dysfunction (POCD) assessment. This study aims to investigate the post-operative cognitive changes among high-risk cardiothoracic patients and establish a standardised approach to post-surgery cognitive assessment.

**Methods:**

This is a prospective cohort study, where cognitive assessments were done 1-day before surgery, at discharge, and during 6 weeks of follow-up. Sample size calculation, accounting for an estimated 20% dropout rate, determined a minimum of 170 subjects were required for the study. Reduction of MMSE score of more than 2.5 was considered as having POCD. Score differences between groups were analysed using *T*-test and analysis of variance (ANOVA), while consistency between tools was analysed using correlation and regression.

**Results:**

A total of 188 patients completed the study, with a POCD prevalence of 20.2% and 6.9% at discharge and at the 6 week follow up, respectively. All cognitive tools show a significant difference between preoperative and postoperative scores. All tests show a significant moderate correlation with MMSE.

**Conclusions:**

In conclusion, it is imperative to employ a battery of cognitive assessments to evaluate cognitive changes comprehensively.

## 1. Introduction 

Cardiothoracic surgery within Malaysia, particularly at our National Heart Institute has undergone notable advancements. The contemporary approach to these life-threatening procedures involves the administration of dexmedetomidine, a promising sedative and analgesic agent proven to have benefits in reducing postoperative complications [1]. Despite this progress, the occurrence of postoperative cognitive dysfunction (POCD) remains a concern, especially among high-risk cardiothoracic patients due to the complex nature of the surgery and associated risks [2]. Given that individuals undergoing these surgeries aspire to resume normal functioning, including the ability for precise decision-making in their professional roles and personal lives, the preservation of cognitive function is an important consideration.

Understanding the exclusive pathogenesis of POCD is still a challenge. Emerging evidence suggests that the inflammatory response plays an important role in its development. Recent studies by Glumac *et al*. [3] (2017) demonstrated that preoperative administration of corticosteroids mitigates the inflammatory response triggered by surgery. Consequently, this intervention led to a reduction in both the severity and incidence of POCD following cardiac surgery [3]. Yazit *et al*. [4] (2023) further confirmed variations in the involvement of inflammatory pathways among different groups of cardiac surgery patients, distinguishing between those with and without POCD. Both studies showed the complexity of the inflammatory response in POCD development [4].

Evaluating the cognitive function of cardiothoracic patients poses a unique challenge, involving tools that are not only reliable but also non-invasive. Additionally, the chosen instruments must embody simplicity to ensure ease for patients recovering from high-risk situations, sparing them from unnecessary fatigue during the assessment process. The Mini-mental State Examination (MMSE) stands out as a comprehensive tool, offering a thorough assessment of multiple cognitive domains (prefrontal cortex; frontal and parietal brain region; occipital and parietal lobes; and hippocampus), including orientation, memory, attention, and language [5]. Its versatility lies in its ability to provide a holistic view of cognitive functioning, making it a widely utilized screening tool [6]. Whereas other assessment tools such as the Trail Making Test (TMT), Digit Span, Digit Symbol Substitution Test (DSST), and Clock Drawing Test (CDT) focus on specific cognitive domains, allowing for a more nuanced examination. The TMT assesses visual attention and task-switching abilities (prefrontal cortex) [7], while Digit Span evaluates working memory (mid-ventrolateral frontal cortex) [8], DSST assesses processing speed [9], and the CDT targets executive function and visuospatial skills [10]. This diversity in tools enables a nuanced understanding of cognitive strengths and weaknesses, contributing to a more refined diagnosis and tailored intervention strategies. Comprehensive cognitive assessment, as facilitated by the MMSE, combined with domain-specific tools, enriches the clinical evaluation of cognitive function, offering a well-rounded perspective on brain health.

Traditionally, the focus in managing cardiothoracic patients has been more on physiological outcomes, but there is growing recognition of the need to assess the outcome in cognitive function. Therefore, there is a compelling argument for the integration of cognitive function evaluation into routine postoperative care. Whilst recommendations exist regarding the selection of tools for cognitive assessment, the abundance of available tools necessitates a thorough evaluation to determine the presence of POCD. Saxena *et al*. [11] (2019) suggested the incorporation of formal perioperative neurocognitive testing to enhance accuracy, reliability and comparability between different cohorts for POCD reporting. Currently, the general definition of POCD is described as either a transient or chronic change in cognitive function that comes after surgery [12]. The focus of this study is on cognitive function assessments, which is in line with current views on complete patient care and acknowledges the importance of cognitive health during recovery. It is imperative to investigate the post-operative cognitive changes among high-risk cardiothoracic patients and establish a standardized approach to post-surgery cognitive assessment. We hypothesise that the cognitive function assessments used in clinical settings are aligned in their reflection of POCD. Therefore, a single approach to assessment is both possible and feasible in a clinical setup.

## 2. Materials and Methods

### 2.1 Study Design and Population

This prospective cohort study focuses on high-risk cardiac surgery patients who received dexmedetomidine as the primary anaesthestic agent. The study was conducted at a single center, specifically, the National Heart Institute, where a cohort of carefully selected patients scheduled for coronary artery bypass surgery (CABG) and/or valve surgery were identified. Inclusion criteria encompassed patients deemed high-risk by overseeing clinicians, possessing proficiency in either Malay or English languages, and not presenting with a diagnosis of dementia. Exclusions were applied to patients with a MMSE score below 24 points, those undergoing off-pump surgery, individuals with severe hepatic impairment, those dependent on dialysis, and pregnant women.

In this study, the consultants reached a consensus on defining high-risk patients within the current clinical context. The subjects comprised consenting adult patients (aged ≥20 years) who underwent cardiac bypass surgery for any of the following reasons:

(1) Multiple valve surgery (≥2 valves ± CABG)

(2) Single valve surgery ± CABG with known moderate to severe pulmonary hypertension

(3) Preoperative mechanical support, including balloon pump or ventricular assist device

(4) Procedures involving the thoracic aorta with planned hypothermic circulatory arrest

(5) Any cardiac surgery patient undergoing a combined operation with an estimated glomerular filtration rate (GFR) of 16–40 mL/min/1.72 $m^{2}$.

Additionally, this includes any post-cardiac surgery patient who, upon admission to the intensive care unit (ICU), was expected to require ventilatory support for more than 48 hours, was receiving very high-dose vasopressor treatment (as determined by the attending intensivist), was on mechanical support other than an elective intra-aortic balloon pump, or was expected to require new dialysis within the first 24 hours after surgery.

Dexmedetomidine infusion commenced at 0.7 mcg/kg/hr (starting at induction of anaesthesia to continue following surgery) or 1 mcg/kg/hr starting postoperatively in ICU, without a loading dose. All patients received standard anaesthesia. All procedures and interventions were provided at the discretion of the anaesthetist in charge. A bispectral index (BIS) target of 40–60% was advisable but not mandatory. Postoperative care was according to standard management as per the intensive care team, including additional sedatives and analgesics, inotropes, vasopressors and ventilatory management.

Sample size determination was done using G*Power software (Version 3.1.9.7, the institute for experimental psychology, Düsseldorf, Germany) based on the F test repeated measures method, with an effect size of 0.25 and a power of 0.95, resulting in a calculated sample size of 142. To account for a potential 20% dropout, the final adjusted sample size was roughly 170. Before their involvement, all patients provided written informed consent, a procedural step adhering to ethical guidelines. The study received ethical approval from the National Heart Institute Ethical Committee (IJNREC/441/2019).

### 2.2 Neurocognitive Assessment

Neurocognitive assessments were done at three (3) timepoints—(1) 1 day before surgery, (2) 7 days post-surgery or at discharge (whichever came first), and (3) at a 6 week post-surgery follow-up. The Malay version of MMSE was adapted during the eligibility screening to eliminate patients with poor cognitive performances at baseline; patients with MMSE scores >24 were recruited. There were 5 tools utilised for cognitive assessment in this study including MMSE, TMT Part A and B, Digit Span Forward and Backward, DSST and CDT. TMT tests are for executive function, task switching and working memory, Digit Span for attention and memory, DSST is for speed and manual dexterity, while CDT is for visuoconstructive abilities. The 1-standard deviation (1 SD) method was used to discriminate the cognitive changes (POCD vs non POCD) post-surgery. According to the method, changes in 1 standard deviation from MMSE baseline scores was considered as cognitive decline. In this study, our 1 SD MMSE score was 2.5, hence patients with a decline of more than 2.5 were considered to have cognitive decline postoperatively.

### 2.3 Statistical Analysis

Statistical analysis was done using SPSS software version 26 (IBM Corp., Armonk, NY, USA). Descriptive analysis using mean ± SD was presented for continuous variables. Frequency analysis was done to see the prevalence of patients with cognitive decline. Normality tests were completed and all variables were normally distributed. Mean comparisons between multiple cognitive tools at 3 (three) time points were analysed using repeated measure analysis of variance (ANOVA). Correlation analysis between cognitive tools was done using Pearson correlation. Multiple linear regression using the enter method was employed to explore the potential association between all cognitive tools for MMSE.

## 3. Results

### 3.1 Characteristics of Study Population

A total of 210 patients consented to be part of the study population. However, only 188 patients successfully completed the whole study (89.5%). Among the 10%; 11 patients deceased during their ICU stay, while an additional 11 patients faced mortality challenges in the immediate post discharge period, before the scheduled 6-week follow-up. Our findings revealed an approximately 10% mortality rate, given the inherently high-risk nature of the patient population under consideration. Nevertheless, the total number of subjects successfully completed the study exceeded the minimum number of sample size required to achieve the desired impact of the study. The characteristics of patients who completed the whole assessments are tabulated in Table 1.

**Table 1.** **Characteristics of our patient population**.

Variables		Frequency (n)	Percentage (%)
Age			
	Below 40	18	9.6
	40–64	122	64.9
	65 and above	48	25.5
Gender			
	Male	122	64.9
	Female	66	35.1
Education level			
	No formal education	3	1.6
	Primary	17	9.0
	Secondary	98	52.1
	Tertiary	70	37.2
Current employment			
	Yes	63	33.5
	No	125	66.5
Household ${\mathrm{income}}^{a}$			
	Low (<MYR 4850)	130	69.1
	Middle (MYR 4850–10,959)	43	22.9
	High (≥MYR 10,960)	15	8.0
Comorbidities (No/Yes)			
	Diabetes	106/82	56.4/43.6
	Hypertension	50/138	26.6/73.4
	Chronic kidney disease	106/82	56.4/43.6
	Hypercholesterolemia	64/123	34.2/65.8
	${\mathrm{Smoking}}^{b}$	100/61	62.1/37.9
Type of surgery			
	CABG only	60	31.9
	CABG + valve	73	38.8
	Valves only	55	29.3

^a^source from Department of Statistic Malaysia. 1 USD = 4.67 MYR. ^b^some values differ due to missing data. CABG, coronary artery bypass graft.

### 3.2 Cognitive Changes in POCD and Non-POCD Patients

The prevalence of POCD in our study was defined by a decrease of more than 2.5 in MMSE score compared to baseline. At discharge, the prevalence of POCD was 20.2% (n = 38), and this percentage decreased during follow-up to 6.9% (n = 13). Mean differences in all cognitive tools were then analysed according to the POCD and non-POCD patients (Table 2).

**Table 2.** **Cognitive changes in cognitive tools at different time points and groups**.

Tools		Baseline	*p*-value	Discharge (POCD, n = 38)	*p*-value	Follow-up (POCD, n = 13)	*p*-value	*p*-value
MMSE		27.5 ± 2.538		26.3 ± 3.979		27.4 ± 3.625		< ${\mathrm{0.001**}}^{a}$
	Non-POCD	27.70 ± 2.514	0.129	27.49 ± 2.687	< ${\mathrm{0.001**}}^{b}$	27.97 ± 2.423	${\mathrm{0.002*}}^{b}$	
	POCD	27.0 ± 2.589		21.42 ± 4.554		19.85 ± 7.290		
TMT-A		52.1 ± 29.267		57.1 ± 37.287		49.9 ± 33.579		${\mathrm{0.004*}}^{a}$
	Non-POCD	52.55 ± 34.274	0.242	53.48 ± 36.043	< ${\mathrm{0.001**}}^{b}$	46.65 ± 25.257	${\mathrm{0.042*}}^{b}$	
	POCD	59.58 ± 27.119		78.16 ± 44.041		97.33 ± 75.993		
TMT-B		121.6 ± 65.750		142.6 ± 79.812		117.7 ± 84.502		< ${\mathrm{0.001**}}^{a}$
	Non-POCD	119.32 ± 68.770	${\mathrm{0.041*}}^{b}$	133.19 ± 79.485	< ${\mathrm{0.001**}}^{b}$	110.29 ± 66.114	${\mathrm{0.019*}}^{b}$	
	POCD	144.55 ± 62.541		188.53 ± 72.114		258.00 ± 185.100		
Digit Span		13.5 ± 3.583		12.5 ± 3.721		13.1 ± 3.736		< ${\mathrm{0.001**}}^{a}$
	Non-POCD	13.62 ± 3.396	0.212	13.03 ± 3.613	< ${\mathrm{0.001**}}^{b}$	13.25 ± 3.692	${\mathrm{0.003*}}^{b}$	
	POCD	12.82 ± 4.132		10.32 ± 3.375		10.00 ± 3.045		
DSST		45.5 ± 15.999		40.2 ± 17.315		47.9 ± 18.931		< ${\mathrm{0.001**}}^{a}$
	Non-POCD	47.78 ± 16.354	< ${\mathrm{0.001**}}^{b}$	42.94 ± 17.427	< ${\mathrm{0.001**}}^{b}$	48.97 ± 18.865	${\mathrm{0.010*}}^{b}$	
	POCD	35.71 ± 11.573		27.46 ± 10.249		33.82 ± 15.217		
CDT		1.87 ± 0.425		1.73 ± 0.602		1.86 ± 0.434		< ${\mathrm{0.001**}}^{a}$
	Non-POCD	1.87 ± 0.430	0.769	1.80 ± 0.532	${\mathrm{0.003*}}^{b}$	1.88 ± 0.387	0.126	
	POCD	1.84 ± 0.495		1.47 ± 0.762		1.50 ± 0.798		

*^a^*p*-value significant at *p* < 0.05 using repeated measure ANOVA. **^a^*p*-value significant at *p* < 0.001 using repeated measure ANOVA. *^b^*p*-value significant at *p* < 0.05 using independent *T*-test. **^b^*p*-value significant at *p* < 0.001 using independent *T*-test. POCD, postoperative cognitive dysfunction; MMSE, Mini-mental State Examination; TMT, Trail Making Test; DSST, Digit Symbol Substitution Test; CDT, Clock Drawing Test; ANOVA, analysis of variance.

The analysis revealed significant differences in cognitive changes in all tools from baseline to the 6-week follow-up (*p* < 0.05). Comparison between POCD and non-POCD groups showed that all cognitive tools were significantly different at discharge and follow-up, except for the CDT. For TMT part A and B, the scores were recorded as the time taken in seconds. Hence, a higher score postoperatively and in the POCD groups denotes a slower time taken to complete the tests. The mean scores for each tool showed significant changes postoperatively and between POCD and non-POCD groups, implying the consistency of those tools with the MMSE. Further analysis was conducted to explore the correlation between all tools and the MMSE, as shown in Table 3.

**Table 3.** **Correlation and regression analysis of cognitive tools towards MMSE**.

Tools	Pearson’s correlation coefficient, *r*	Regression, *$r^{2}$*	Crude OR (CI)	*p*-value	Adjusted OR (CI)	*p*-value
TMT-A	–${\mathrm{0.434*}}^{a}$	0.188	–0.041 (–0.048 to –0.034)	<0.001**	–0.057 (–0.014 to 0.003)	0.236
TMT-B	–${\mathrm{0.508*}}^{a}$	0.258	–0.023 (–0.026 to –0.020)	<0.001**	–0.126 (–0.010 to –0.001)	0.023*
Digit Span	${\mathrm{0.468*}}^{a}$	0.219	0.435 (0.369 to 0.501)	<0.001**	0.252 (0.169 to 0.299)	<0.001**
DSST	${\mathrm{0.541*}}^{a}$	0.293	0.102 (0.089 to 0.115)	<0.001**	0.269 (0.034 to 0.68)	<0.001**
CDT	${\mathrm{0.350*}}^{a}$	0.123	2.321 (1.823 to 2.820)	<0.001**	0.142 (0.486 to 1.392)	<0.001**

${*}^{a}$*p*-value significant at *p* < 0.05 using Pearson’s correlation coefficient. *p-value significant at *p* < 0.05. **p-value significant at *p* < 0.001. MMSE, Mini-mental State Examination; TMT, Trail Making Test; DSST, Digit Symbol Substitution Test; CDT, Clock Drawing Test; OR, odds ratio.

TMT, Digit Span and DSST showed a moderate correlation with MMSE, whereas the CDT had a poor correlation, despite having a statistically significant correlation in all tests. The result of a multiple linear regression analysis revealed that TMT part B, Digit Span, DSST and CDT were statistically significant, implying a close association with MMSE.

## 4. Discussion

This study has successfully elucidated the correlation between multiple cognitive assessments to thoroughly assess cognitive changes after surgery. The low prevalence of POCD found in the study population that homogenously received dexmedetomidine together with the clinical importance of a 2.5-point decrease in MMSE scores are the highlighted findings in this study. It has been found that the association between various types of sedation and POCD is multifaceted [13]. Hence, it is imperative to explicitly outline the standardization of sedation administered to the subjects in this study whilst discussing the prevalence of POCD within this patient cohort.

The comprehensive evaluation of cognitive function pre- and post-surgery, incorporating a diverse array of cognitive assessment tools such as the MMSE, TMT, Digit Span, DSST, and CDT, establishes a robust foundation for delineating the intricate landscape of cognitive changes in the perioperative period. This multifaceted approach allows for a comprehensive exploration of cognitive domains and accentuates the sensitivity of each tool to specific aspects of cognitive function. The MMSE, with its broad coverage of cognitive domains, provides a holistic snapshot of cognitive health, while the supplementary tools, such as the TMT, Digit Span, DSST, and CDT, contribute unique insights into executive function, working memory, processing speed, and visuospatial abilities. This comprehensive assessment approach is in line with recent literature advocating for a multidimensional evaluation strategy to more accurately capture the complex and heterogeneous nature of postoperative cognitive changes [14, 15].

The nuanced understanding of cognitive alterations, as facilitated by this multi-tool approach, is particularly pertinent in the context of cardiothoracic surgeries, where the intricate interplay of physiological and psychological factors can influence cognitive outcomes. Moreover, the identification of cognitive domains affected by surgery such as memory, attention, executive functions, and processing speed leads to challenges in recalling information, focusing, problem-solving, and processing efficiently. Targeted post-surgery interventions, including memory enhancement techniques, attention training, executive function programs, cognitive therapy, physical exercise, and mindfulness practices, are crucial for recovery. By identifying affected domains and implementing personalised interventions, healthcare providers can optimise patient well-being, ensuring a smoother postoperative recovery process and enhancing overall outcomes [16].

Based on this observational study, a relatively low prevalence of POCD and the decline from an immediate POCD rate of approximately 20% to 7% after a 6-week period, offers compelling insight into the dynamic and transient nature of cognitive changes following surgery. These findings align with current literature emphasizing that cognitive alterations post-surgery may often exhibit a temporary trajectory, with recovery occurring over time [17, 18]. The documented decrease in POCD rates over the 6 weeks suggests a potential adaptive capacity of the central nervous system, highlighting the need for an understanding of the evolving cognitive landscape in the postoperative phase. Nonetheless, the sobering acknowledgement of an approximate 10% mortality rate among subjects before the 6-week flow-up serves as a reminder of the multifaceted nature of surgical outcomes. Findings from this study emphasise the interconnectedness of cognitive and clinical domains in the evaluation of surgical outcomes, urging a shift towards an integrative patient-centreed approach [16, 19]. The approach enhances the precision of outcome assessments and facilitates tailored interventions that address both cognitive and clinical aspects, ultimately contributing to holistic patient care.

The statistically significant correlations observed between the MMSE and other cognitive assessment tools, including the TMT, Digit Span, DSST, and CDT, underscore the presence of a strong linear relationship among these measures. Findings from this study validate the individual tools but also emphasize their collective efficacy in capturing the broader spectrum of cognitive function. The identified correlations contribute to the converging evidence across diverse assessment methods, reinforcing the reliability and interrelated nature of these cognitive instruments [15]. Moreover, the observed linear regression provides additional support for the proposition that changes in MMSE scores correspond to changes in performance on specific cognitive tasks. This alignment further strengthens the potential of MMSE in reflecting alterations across various cognitive domains, offering a comprehensive overview of cognitive changes postoperatively. Such consistent relationships between MMSE and other cognitive tools provide a cohesive framework for assessing and interpreting cognitive dynamics following surgery.

In addition, the application of 1 SD method to determine POCD in this study introduces the concept that the score lowering by 2.5 is a significant change post-operatively which indicates significant cognitive decline. The significant mean differences observed in TMT, Digit Span, DSST, and CDT between POCD and non-POCD groups can be determined by the selected reduction score (–2.5) validates the clinical utility of the selected score. Particularly noteworthy is the identification of a 2.5-point reduction in MMSE scores as a meaningful indicator of cognitive decline postoperatively. This reduction serves as a practical threshold for identifying patients at risk for cognitive changes, facilitating early intervention and personalised postoperative care strategies [14, 16].

## 5. Conclusions

In conclusion, this study underscores the importance of employing a battery of cognitive assessments to comprehensively evaluate cognitive changes post-surgery. The observed low prevalence of POCD, coupled with the robust correlations and clinical significance of a 2.5-point reduction in MMSE scores, contributes to the refinement of postoperative cognitive assessment strategies, enhancing the precision of identifying and addressing cognitive changes in surgical populations.

In the current setting, one limitation encountered was the inability to effectively stratify groups by age. Previous studies have identified age as a significant factor influencing POCD with specific appropriate cognitive assessment tools. It is recommended that future research delve into this factor by examining different age groups. Additionally, while this study may demonstrate the benefits of using a single method for diagnosing POCD, it does not address the tool’s feasibility in diagnosing long-term POCD. Future research directions may incorporate this aspect. In conjunction with cognitive assessment tools, future direction may include advanced neuroimaging techniques (such as magnetic resonance imaging (MRI) or positron emission tomographic (PET) scans) to detect structural and functional brain changes associated with POCD. This approach aims to explore the correlation between objective and subjective measures in assessing cognitive decline outcomes.

## Data Availability

All data are available in this manuscript.
